# Advances in Interfacial Engineering and Structural Optimization for Diamond Schottky Barrier Diodes

**DOI:** 10.3390/ma18153657

**Published:** 2025-08-04

**Authors:** Shihao Lu, Xufang Zhang, Shichao Wang, Mingkun Li, Shuopei Jiao, Yuesong Liang, Wei Wang, Jing Zhang

**Affiliations:** 1 School of Integrated Circuits, North China University of Technology, Beijing 100144, China; shihaolu736@gmail.com (S.L.);; 2 Key Laboratory of Physical Electronics and Devices, Ministry of Education, School of Electronic Science and Engineering, Xi’an Jiaotong University, Xi’an 710049, China

**Keywords:** diamond, MS SBDs, MIS SBDs

## Abstract

Diamond, renowned for its exceptional electrical, physical, and chemical properties, including ultra-wide bandgap, superior hardness, high thermal conductivity, and unparalleled stability, serves as an ideal candidate for next-generation high-power and high-temperature electronic devices. Among diamond-based devices, Schottky barrier diodes (SBDs) have garnered significant attention due to their simple architecture and superior rectifying characteristics. This review systematically summarizes recent advances in diamond SBDs, focusing on both metal–semiconductor (MS) and metal–interlayer–semiconductor (MIS) configurations. For MS structures, we critically analyze the roles of single-layer metals (including noble metals, transition metals, and other metals) and multilayer metals in modulating Schottky barrier height (SBH) and enhancing thermal stability. However, the presence of interface-related issues such as high densities of surface states and Fermi level pinning often leads to poor control of the SBH, limiting device performance and reliability. To address these challenges and achieve high-quality metal/diamond interfaces, researchers have proposed various interface engineering strategies. In particular, the introduction of interfacial layers in MIS structures has emerged as a promising approach. For MIS architectures, functional interlayers—including high-k materials (Al_2_O_3_, HfO_2_, SnO_2_) and low-work-function materials (LaB_6_, CeB_6_)—are evaluated for their efficacy in interface passivation, barrier modulation, and electric field control. Terminal engineering strategies, such as field-plate designs and surface termination treatments, are also highlighted for their role in improving breakdown voltage. Furthermore, we emphasize the limitations in current parameter extraction from current–voltage (I–V) properties and call for a unified new method to accurately determine SBH. This comprehensive analysis provides critical insights into interface engineering strategies and evaluation protocols for high-performance diamond SBDs, paving the way for their reliable deployment in extreme conditions.

## 1. Introduction

Diamond possesses an ultra-wide bandgap (5.47 eV), a high breakdown electric field (>10 MV/cm), high carrier mobility (with hole mobility at 3800 cm^2^/V·s and electron mobility at 4500 cm^2^/V·s), and high thermal conductivity (>2000 W/m·K) [1,2,3]; therefore, diamond is considered as an ideal material for power-electronics applications [4,5,6]. Diamond power devices have been extensively studied and among them, diamond Schottky barrier diodes (SBDs) have attracted significant attention due to their relatively simple structure and superior characteristics, including low forward voltage drop, fast response speed, and low power consumption [7,8,9].

In diamond SBDs, the structure most commonly employed is the metal–semiconductor (MS) configuration. For p-type diamond, oxygen-terminated diamond (O-diamond) is critical to achieve rectifying contacts, as it typically exhibits positive electron affinity (PEA), which promotes the formation of Schottky barrier height (SBH) [10,11]. In contrast, hydrogen-terminated diamond (H-diamond) with its negative electron affinity (NEA) mainly induces ohmic behavior [12,13]. In diamond MS SBDs, the choice of metal plays a crucial role in determining the electrical properties of the device. Generally, single-layer metals and multilayer metals are used as Schottky metal contacts. Single-layer metals include noble metals such as Gold (Au [14,15,16,17,18,19,20,21,22,23]), Platinum (Pt [24,25]), and Palladium (Pd [26]), as well as transition metals like Molybdenum (Mo [27,28,29]), Nickel (Ni [30,31]), Chromium (Cr [32]), and other metals like Aluminum (Al [33]) and Tungsten Carbide (WC [34,35]). In addition, multilayer metals composed of several layers, such as Zr/Pt/Au, Ni/Au, and Au/Pt/Ni, can provide better thermal stability [36].

Despite these advantages, diamond MS SBDs suffer from critical challenges. One key issue is the high density of interface states at the metal/diamond contacts, which often leads to Fermi level pinning [37,38,39]. Consequently, this problem results in poor control of SBH, thus resulting in increased reverse leakage current, thereby restricting the applicability of diamond SBDs in harsh environments [25,40]. To overcome these challenges, researchers have proposed approaches to improve the MS interface quality. They have attempted to insert an interlayer between the metal and diamond, forming a metal–interlayer–semiconductor (MIS) SBD structure. This configuration effectively passivates the interface states, while enabling barrier modulation via interface dipole formation or shielding effects [41]. Additionally, it suppresses the diffusion of metal atoms at the metal/diamond interface, thereby enhancing the thermal stability and long-term reliability of the devices [42,43,44]. The presence of the MIS structure provides a more flexible and multidimensional structural design solution for enhancing the performance of diamond SBDs.

In this work, we systematically reviewed the research progress of the diamond SBDs. We discussed the impacts of metal selection and interlayer material selection on the diamond MS and MIS device performances, respectively. Specifically, this work was organized as follows: Section 2 discusses the fundamental physical properties and extraction of key parameters of diamond SBDs based on current–voltage (I–V) and capacitance–voltage (C–V) characteristics, and further incorporates a discussion on barrier inhomogeneity modeling and temperature dependence. Section 3 summarizes recent advances in diamond MS SBDs, with particular focus on the roles and mechanisms of different metal contacts, including single-layer metals (noble metals, transition metals, and other metals) and multilayer metals in enhancing device performances. Section 4 focuses on recent research advances in diamond MIS SBDs, emphasizing the mechanisms of different functional interlayers, including interface passivation interlayers, barrier modulation by low-work-function interlayers, and terminal electric field control interlayers. In Section 5, we discuss how factors may lead to discrepancies between the measured and true SBH, suggesting the need for more methods to accurately extract parameters.

## 2. Diamond Schottky Barrier Diodes (SBDs)

### 2.1. Energy Band Contacts in Diamond SBDs

**Figure 1:**
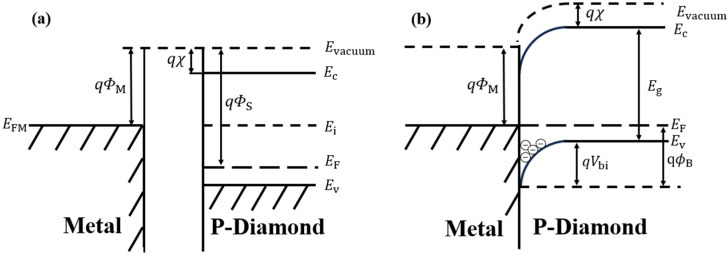
The energy band diagrams (**a**) before and (**b**) after metal/p-type diamond contact (*W*_M_ < *W*_S_).

The rectifying behavior of diamond SBDs originates from the Schottky barrier formed at the metal–semiconductor interface. Taking p-type diamond as an example, its Fermi level is lower than that of most common metals in the initial state. When the metal contacts p-type diamond, the energy band diagram would redistribute to achieve thermal equilibrium. Holes flow from the diamond to the metal, causing electron accumulation on the diamond contact side. As a result, the diamond energy band bends downward, ultimately aligning the Fermi levels of both sides. Consequently, the SBH can be formed between the metal and diamond. The energy band diagrams before and after metal/diamond contact are shown in Figure 1a and Figure 1b, respectively. The downward bending of the energy bands forms the built-in potential (*V*_bi_). According to the Schottky–Mott theory [45], the Schottky barrier height (*Φ*_B_) ideally results from the difference between the metal work function (*W*_M_) and the electron affinity of diamond (*χ*), as expressed as follows:

(1) $$\Phi_{B}=\phi_{M}-\chi .$$

### 2.2. Extraction of Key Parameters in SBDs

In an ideal diamond SBD, the forward current–voltage (I–V) relationship follows the thermionic emission (TE) theory [46,47], and it can be expressed by the following equation [48]:

(2) $$I=I_{0}(e^{\frac{qV_{D}}{nkT}}-1),$$

where *I*_0_ is the saturation current density, *V*_D_ is the voltage drop across the device, *n* is the ideality factor, *k* is the Boltzmann constant, and *T* is the absolute temperature, respectively. According to the TE theory, holes in p-type diamond are thermally excited over the Schottky barrier and injected into the metal [49]. When a forward bias is applied to the SBDs, the barrier between the metal and diamond is effectively reduced, allowing more majority carriers (holes) to cross the barrier and generate current.

In Schottky diodes, the saturation current density *I*_0_ is directly related to the *Φ*_B_. According to the thermionic emission theory, *I*_0_ can be expressed by the following equation:

(3) $$I_{0}=AA^{*}T^{2}e^{-\frac{q\phi_{B}}{kT}}.$$

Here, *A* is the effective area of the diode, and A* is the Richardson constant. It is clear that the saturation current *I*_0_ decreases as the *Φ*_B_ increases, thus affecting the reverse current and breakdown voltage of the diode.

In addition, the impact of series resistance (*R*_S_) on I–V characteristics of diamond SBDs has to be considered [50,51,52]. When the current flows under a certain voltage, the voltage across the diode is *V*_D_ = *V* − *IR*_S_. When *V*_D_ > 3*kT*/*q*, Equation (2) can be expressed in the following [53]:

(4) $$I=I_{0}e^{\frac{q(V-IR_{s})}{nkT}}.$$

Based on Equations (3) and (4), the applied voltage can be deduced as follows:

(5) $$V=\frac{nkT}{q}\ln\frac{J}{A^{*}T^{2}}+{n\phi }_{B}+JAR_{s},\;$$

Note that *J* is the current density denoted as *I*/*A*. Differentiating Equation (5) with respect to *J* and rearranging terms, we obtain the following:

(6) $$\frac{dV}{dlnJ}=\frac{nq}{kT}+JAR_{s}.$$

This approach allows the extraction of important parameters from I–V, like *Φ*_B_ and *n*, which are essential for characterizing the SBDs’ performance. The accuracy of these extracted parameters is critical for optimizing the diode’s electrical characteristics. However, practical diamond SBDs exhibit deviations from the ideal TE model. It has been reported that a native interlayer is generally formed between metal and diamond [54]. The voltage drop across the device would be affected by the insertion of the interlayer, and thus, the ideality factor n would be modulated. Typically, *n* is greater than 1 due to the impacts of interface states or other defects at the metal–semiconductor junction [55,56].

To complement key parameters, C–V test is particularly important because it can assess interface state density (*N*_SS_) and offer critical information on the quality of the MS interface. Specifically, the C–V method measures the variation of the depletion region capacitance as a function of the applied bias, and its relationship can be expressed as follows [43]:

(7) $$C=\frac{A\varepsilon_{s}\varepsilon_{0}}{W_{D}}=A\sqrt{\frac{q\varepsilon_{s}\varepsilon_{0}N_{A}}{2(V_{bi}-\frac{kT}{q}-V)}}.$$

Furthermore, the *N*_SS_ can be quantitatively calculated by the following [57]:

(8) $$N_{SS}=\frac{1}{q}\left[\frac{\varepsilon_{i}\varepsilon_{0}}{\delta }\left(n\left(V\right)-1\right)-\frac{\varepsilon_{s}\varepsilon_{0}}{W_{D}}\right],$$

where *ε*_i_ is the interface dielectric constant, *δ* is the interfacial layer thickness, and *n*(V) can be extracted from Equation (4). This expression provides a direct means to estimate the density of interface states from combined I–V and C–V measurements, thereby enabling a deeper understanding of the interfacial properties and their impact on device performance.

### 2.3. Barrier Inhomogeneity Modeling and Temperature Dependence in Diamond SBDs

In an ideal metal–semiconductor interface, the Schottky barrier height (SBH) is determined by the metal work function and semiconductor electron affinity. However, in real devices, SBHs often exhibit significant spatial inhomogeneity, resulting from metallic diffusion spikes into the semiconductor, the roughness of the interface, and local barrier lowering by image forces [58,59]. These inhomogeneities lead to non-ideal behavior such as discrepancies of SBH between I–V and C–V, high ideality factors, and curved Richardson plots.

To account for these effects, Werner and Güttler proposed a widely adopted model where the spatial distribution of local SBHs is represented by a Gaussian distribution with a mean barrier height $\bar{\phi_{B}}$ and a standard deviation σ_S0_. Based on this model, the effective barrier heights extracted from I–V measurements at zero bias are expressed as follows:

(9) $$\phi_{B0}^{I}=\bar{\phi_{B0}}-\frac{q\sigma_{S0}}{2kT},$$

This implies that the apparent SBH is expected to depend strongly on temperature, explaining the downward curvature of J/T^2^ (from Equation (5))–T^−1^ plots often observed in experimental data. Such plots are often used to deduce the SBH from the slope. In contrast, the barrier extracted from C–V measurements corresponds to the mean barrier and is relatively temperature independent [60]:

(10) $$\phi_{B0}^{C}\equiv \bar{\phi_{B0}}.$$

Similarly, the ideality factor exhibits a temperature-dependent variation. *ρ*_1_, and *ρ*_2_ are the voltage coefficients.

(11) $$n^{-1}\left(T\right)-1=-\rho_{1}+\frac{q\rho_{2}}{2kT}.$$

Note that it is difficult to distinguish inhomogeneity effects from other sources, such as interface degradation or trap states, just relying on single-temperature I–V or C–V measurements. Thus, multi-temperature measurements are essential for reliable and physically meaningful parameter extraction.

To further refine inhomogeneity analysis, alternative models such as Tung’s model and the p-diode model have been developed [61,62]. These models aim to more realistically capture spatially nonuniform transport, particularly in devices where multiple transport paths coexist. Despite their increased complexity, all these methods originate from the parallel conduction theory, in which the total current is treated as a superposition of currents flowing through multiple regions with distinct local barrier heights [63].

In practice, C–V characterization is often affected by nonidealities such as the presence of an interlayer between the metal and the semiconductor, interface states at the interlayer and semiconductor interface [64,65,66], imperfect ohmic back-contacts (and therefore minority carrier injection effect) [67,68] and the series resistance of the neutral region of the SBDs [69,70,71]. These issues result in significant deviation from ideal capacitance behavior, particularly under high-frequency conditions. In contrast, I–V measurements are typically more robust against measurement noise. Therefore, I–V measurement is the predominant method for barrier extraction in the literature.

## 3. Research Progress of Diamond MS SBDs

**Figure 2:**
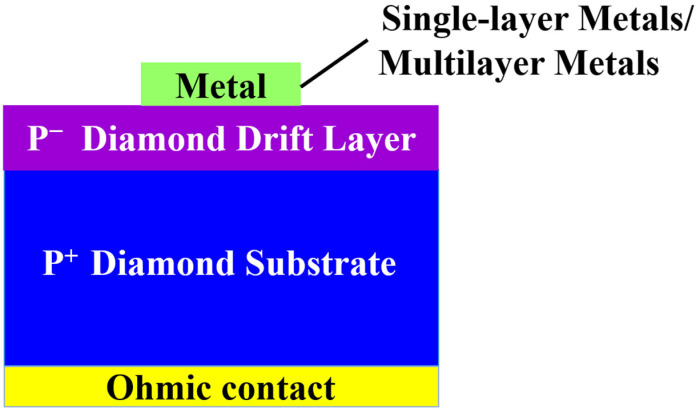
Schematic of vertical p-diamond SBDs with single-layer or multilayer metals.

Diamond MS SBDs have been extensively studied in recent years, which can be categorized into two mainstreams: one is a single-layer metal/diamond structure, and the other is a multilayer metal/diamond structure, as illustrated in Figure 2. The following section examines how different metal contact designs modify the SBH, evaluating both single-layer and multilayer approaches.

### 3.1. Single-Layer Metals/Diamond SBDs

Single-layer metal contacts have been commonly employed in diamond SBDs due to their relatively simple structure and effective performance in specific applications. The choice of metal significantly impacts the SBH, *n*, and reverse leakage current [72]. Three types of single-layer metals commonly used in diamond SBDs are noble metals, transition metals, and other metals.

#### 3.1.1. Nobel Metals/Diamond SBDs

Noble metals, such as Au, Pt, and Pd, have been widely employed as the Schottky metals to fabricate diamond Schottky diodes. Noble metals have excellent physicochemical stability and high work function [73,74,75].

**Figure 3:**
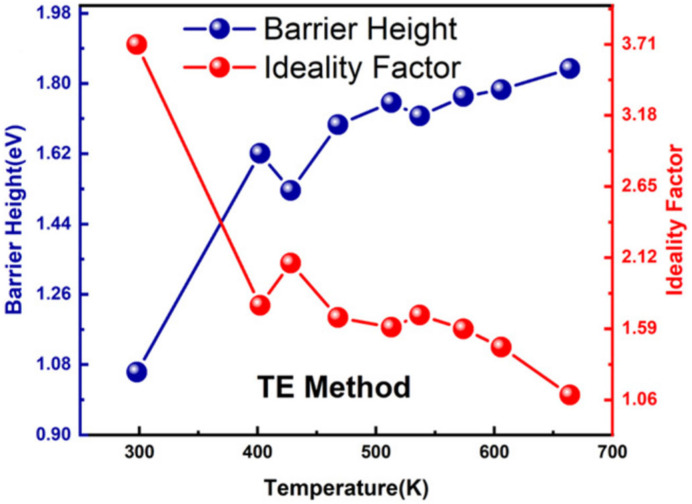
Temperature-dependent *Φ*_B_ and *n* of Au/diamond SBDs [77].

In 2014, Teraji et al. [76] conducted research on the formation of Schottky contacts by Au on O-diamond and found that the SBH of Au/diamond SBDs is relatively high and less affected by O-diamond surface defects, thus enabling more stable electrical performance. In addition, Liang et al. [77] developed high-sensitivity diode temperature sensors (DTSs) based on pseudo-vertical diamond SBDs with Au. Their study demonstrated that the fabricated DTSs exhibited ultrahigh sensitivities of 22.68 mV/K (298–468 K) and 9.92 mV/K (468–664 K) at a current of 1 × 10^−3^ A, which are among the highest reported values for wide bandgap semiconductor-based DTSs. As illustrated in Figure 3, the temperature-dependent SBH and n extracted from I–V properties revealed key interfacial properties: SBH increased from 1.06 eV at 298 K to 1.84 eV at 664 K, while *n* decreased from 3.71 to 1.09 over the same range. This inverse correlation between *Φ*_B_ and *n* directly confirmed the inhomogeneous nature of the Schottky contact, with carrier transport transitioning from multi-path mechanisms (e.g., tunneling [78,79]) at low temperatures to dominant thermionic emission at high temperatures [59,80]. Thereby, the high sensitivity of the work was attributed to the inhomogeneous Schottky contact.

Ueda et al. [30] fabricated Ag/diamond SBDs and investigated the high-temperature reliability of these devices. The results showed that the rectification ratio of Ag/diamond SBDs remained greater than 10^4^ at 600 °C, and even at 750 °C, the rectification ability was maintained above 10, demonstrating excellent thermal stability. In 2024, Abdelrahman et al. [81] developed Pt/diamond SBDs, where SBH is 1.64 eV and breakdown voltage is 453 V. The corresponding Baliga power factor exceeded 5 MW/cm^2^, placing it at the forefront of similar structures and highlighting the advantages of platinum materials in high-power device applications.

Numerous studies have demonstrated that noble metal contacts, such as Au, Pt, and Pd, not only facilitate the formation of high SBH and high rectification ratios, but also exhibit superior thermal stability [82]. High work function and strong chemical inertness of noble metals, such as Au, Pt, and Pd, contribute to the formation of stable metal/diamond interfaces. The use of noble metals in diamond SBDs enhances the thermal reliability and operational stability of diamond-based SBDs, as evidenced in Ru/diamond [83].

#### 3.1.2. Transition Metals/Diamond SBDs

Koné et al. [84] compared the impact of the different transition metals of W and Ni on the electronic performance and stability under high temperatures (~525 K) of the SBDs. The *n* is extremely close to unity (*n* ≈ 1) for W and Ni contacts and is almost insensitive to temperature increase. Besides, Ni/diamond SBDs displayed extremely low reverse leakage current and high current density, showing their potential for high-temperature applications. For the W/diamond SBDs, although the current density was somewhat lower compared to Ni, the W contacts still showed remarkable thermal stability, with minimal reverse leakage current over the entire temperature range. In addition, VanderWeide and Nemanich fabricated titanium (Ti)/diamond SBDs and found that the interface between Ti and diamond tends to form a carbide layer of TiC [85], which degrades the stability of the Schottky contact and causes instability in the SBH. However, transition metals such as Ni and W rarely undergo this reaction, possessing higher chemical stability, thus maintaining better thermal stability and electrical performance at high temperatures.

**Figure 4:**
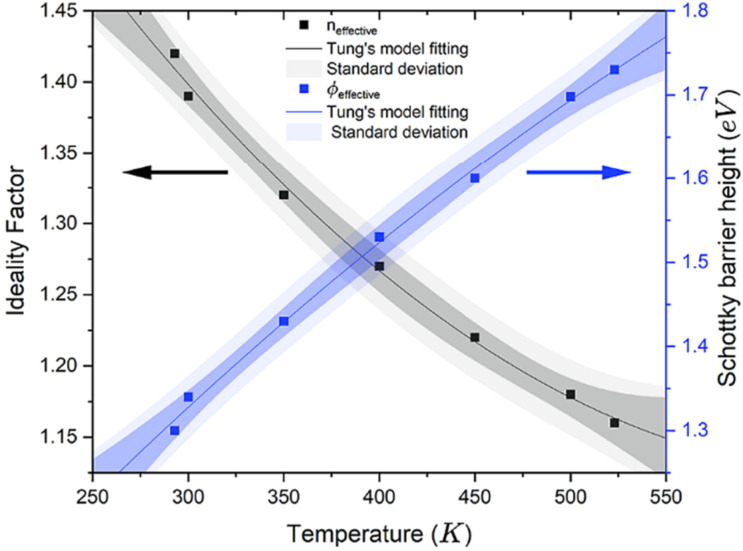
The variation of diode parameters (*n* and *Φ*_B_) as a function of temperature for Mo/diamond SBDs with electrode diameter of 65 μm [81].

In 2016, Eon et al. [86] constructed an O-diamond SBD with Zirconium (Zr), which is easily oxidized, and the device demonstrated excellent device characteristics. The Zr/p-diamond diode achieved a forward current density, with the value up to 103 A/cm^2^ at room temperature, and a breakdown field of 7.7 MV/cm. The corresponding Baliga’s figure of merit (BFOM) reached 244 MW/cm^2^, which was one of the highest values reported at the time. Meanwhile, the Zr/diamond contact maintained good thermal stability after annealing at 450 °C, with the SBH decreasing from an initial value of 1.88 eV to 1.0 eV, indicating the rectification performance had no significant degradation. Besides the SBD developments on homoepitaxial diamond substrates summarized above, very recently, Abdelrahman et al. deposited Mo and Cr on a heteroepitaxial diamond substrate and explored the effect of different electrode sizes on the performance of the diamond SBDs [81]. The results showed that as the metal contact size increased, the SBH of Mo and Cr contacts significantly increased, which may be related to the effects of Schottky barrier inhomogeneity [87]. Additionally, the reduction in the on-resistance of the diodes with smaller contact sizes may be attributed to localized electric field effects promoting impurity ionization, alongside the geometric scaling of parasitic resistances [88]. Furthermore, the temperature dependence of the effective SBH and *n* from Mo are shown in Figure 4. As the temperature increases, the SBH tends to rise, and the ideality factor decreases. According to Tung’s model, carriers are able to get energy and overcome the high SBH as the temperature increases [89].

#### 3.1.3. Other Metals/Diamond SBDs

In addition to noble and transition metals, certain non-typical metals or metal compounds have also been employed as metal contacts for diamond SBDs in recent years. Though these materials do not possess the ability to control carrier transport of traditional gold nor the excellent thermal stability of transition metals, they exhibit unique advantages of low power integration and high temperature reliability. Metals such as main group metals (such as Al) and carbide metals (such as WC) are categorized as “other metal systems” and play an irreplaceable role in the practical development of devices.

In 2012, Koné et al. evaluated the contact performance of aluminum (Al) on O-diamond surfaces [84]. Although Al exhibited good rectifying behavior at room temperature, the reverse leakage current increased sharply at high temperatures (>575 K). Al tends to form reactive layers such as Al_4_C_3_ at the interface, which weakens the control over the SBH [90]. In 2017, Teraji et al. investigated the feasibility of WC acting as the Schottky contacts for O-diamond SBDs [91]. They studied the impact of electrode size on the Schottky barrier height and reverse leakage current of the SBDs. The results showed that both parameters have no significant change, indicating excellent electrical properties and interface consistency. With its low reverse current density (~10^−9^ A/cm^2^) and stable barrier structure, WC has become a promising contact material that combines excellent electrical performance and mechanical stability [34].

**Figure 5:**
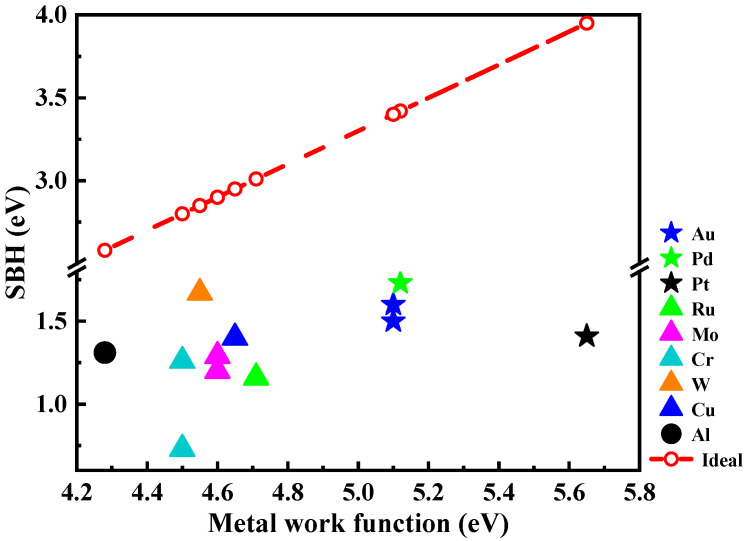
The state of the art of SBH from single metal/p-diamond Schottky contacts. The red line represents the ideal Schottky–Mott trend.

We summarized the reported metal work function and the corresponding SBH, as shown in Figure 5 [19,24,76,81,83,84,86,92]. According to the Schottky–Mott model, the SBH should theoretically increase linearly with the metal work function [45]. In Figure 5, the dashed line represents the ideal SBH trend calculated using Equation (1), where *χ* is the electron affinity of O-diamond (χ = 1.7 eV [93]). The actual SBHs of different metal/p-diamond contacts do not follow this ideal trend. This discrepancy is primarily attributed to Fermi level pinning effects at the metal/diamond interface, which can affect the SBH directly [94,95,96]. A comparative study by Abdelrahman et al. [97] on Au, Ag, W, Pt, and Pd contacts revealed that the measured SBHs deviate from the ideal trend expected from their work functions, highlighting the complex interfacial interactions of SBH formation. These findings underscore the importance of considering interface states when designing high-performance diamond SBDs with various metal contacts. This deviation highlights that SBH is not only solely governed by the intrinsic of the metal and diamond, but also affected by the complex interfacial conditions [98].

### 3.2. Multilayer Metals/Diamond SBDs

**Figure 6:**
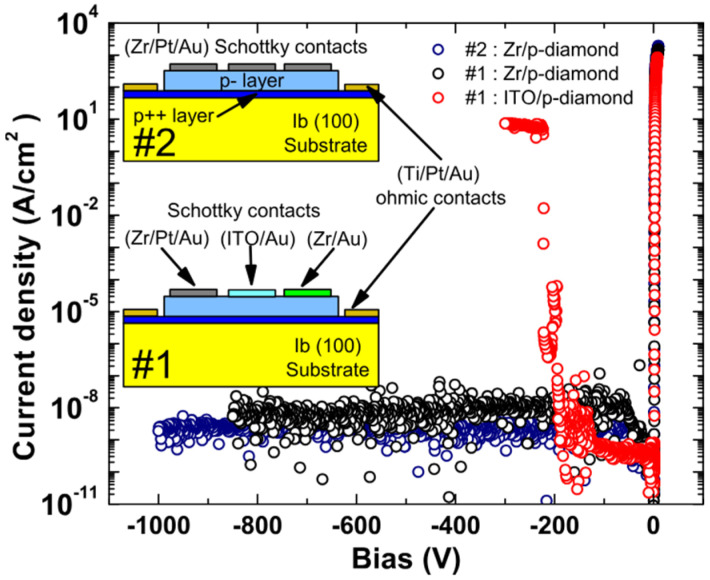
Reverse I–V characteristics of Zr/p-diamond and ITO/p-diamond Schottky SBDs [106].

Although noble and transition metals have been widely used in diamond SBDs, single-layer Metal structures still face many limitations in high-temperature and high-power operating environments, such as electrical property degradation caused by metal diffusion [99], limited Schottky barrier height [100], and restricted control over carrier injection paths [101,102,103,104,105]. To overcome these challenges, researchers have proposed the concept of multilayer-metal contacts. In 2014, Traoré et al. [106] employed Zr/Pt/Au as the contact metal to fabricate diamond SBDs, where the I–V demonstrated a remarkable forward current density of 10^3^ A/cm^2^ at 6 V bias, significantly surpassing ITO-based devices. The bottom Zr layer forms a zirconium oxide (ZrO_2_) thin film with the O-diamond surface, providing a uniform barrier contact foundation. The middle Pt layer, as an inert metal, effectively suppresses further oxidation of the metal, while the top Au layer ensures excellent conduction performance. The electrical characteristics are shown in Figure 6. After annealing at 450 °C, the structure still maintains low reverse leakage current (<1.3 × 10^−9^ A/cm^2^) and high breakdown field (7.7 MV/cm), with a BFOM value of 244 MW/cm^2^, exhibiting excellent electrical characteristics. It is obvious that this structure demonstrates the synergistic effects of each metal.

Additionally, to address the issue of current inconsistency in large-area devices, Nikolenko et al. [107] employed a multi-metal structure of Au/Pt/Ni. In the work, the n was stabilized in the range of 1.1~1.3, and the reverse leakage current was also noticeably reduced (about 10^−11^ A). The introduction of Pt stabilized electron transport between the electrodes and reduced hysteresis effects [108]. Erlbacher et al. [109] used a Ti/Au bilayer structure as the metal contact in their diamond SBDs for potential high-temperature power applications. Ti serves as an adhesive layer to form a Schottky barrier with diamond, and Au provides excellent conductivity and interface stability. This structure achieved a breakdown field of up to 1.75 MV/cm and stable rectifying characteristics, even without complex diffusion barrier layers. This demonstrates that an appropriate combination of metals and interface matching can achieve excellent electrical performance even with a simple metal stacking scheme. The key lies in optimizing the balance on interface reactivity control [110,111].

In summary, the selection of metal contacts—whether through single-layer metals or multilayer metals configurations—has proven effective in modulating SBH, reducing *n*, and enhancing electrical properties. Noble metals generally possess higher work functions and good interface matching with diamond, forming stable and high-barrier contacts. Transition metals strike a balance between cost, process compatibility, and high temperature performance. Other metals (such as main group metals and metal carbides) offer extended pathways for specialized application needs.

However, most of these studies primarily focus on optimizing the electrical parameters extracted from I–V, such as SBH and *n*, while the deeper physical mechanisms at the metal/diamond interface remain insufficiently understood [112]. In particular, limited attention has been paid to critical interfacial factors such as interface states and Fermi-level pinning.

## 4. Research Progress of Diamond MIS SBDs

While previous sections have primarily focused on optimizing SBH and *n* in diamond MS SBDs, it is increasingly recognized that electrical properties are also influenced by the quality of the metal/diamond interface [113]. In particular, the presence of *N*_SS_ induced by lattice mismatch and vacancies at the metal/diamond interface would adversely affect the quality of the interface [114,115]. *N*_SS_ would lead to Fermi-level pinning at high densities and create undesirable leakage current paths [116,117].

**Figure 7:**
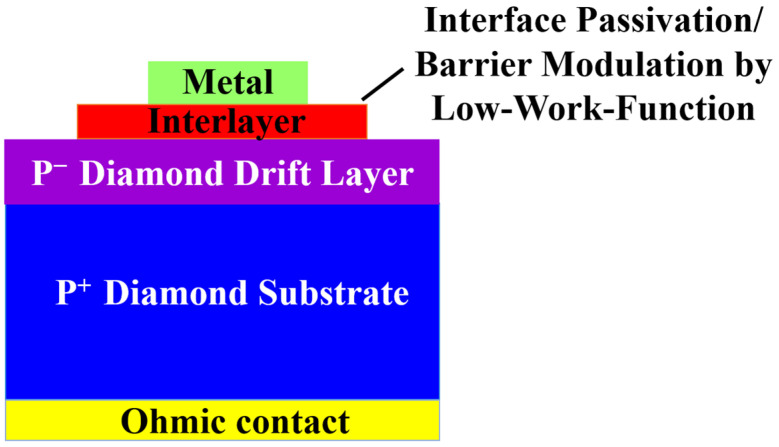
The structure of the MIS SBDs.

To further resolve the issues of high *N*_SS_ and Fermi-level pinning, researchers have attempted a new structure of MIS SBDs, as shown in Figure 7. By introducing a layer with specific functions between the metal and diamond, *N*_SS_ can be effectively reduced, creating spatial isolation between the metal and semiconductor direct contact [118,119,120].

### 4.1. Interface Passivation Interlayers

**Figure 8:**
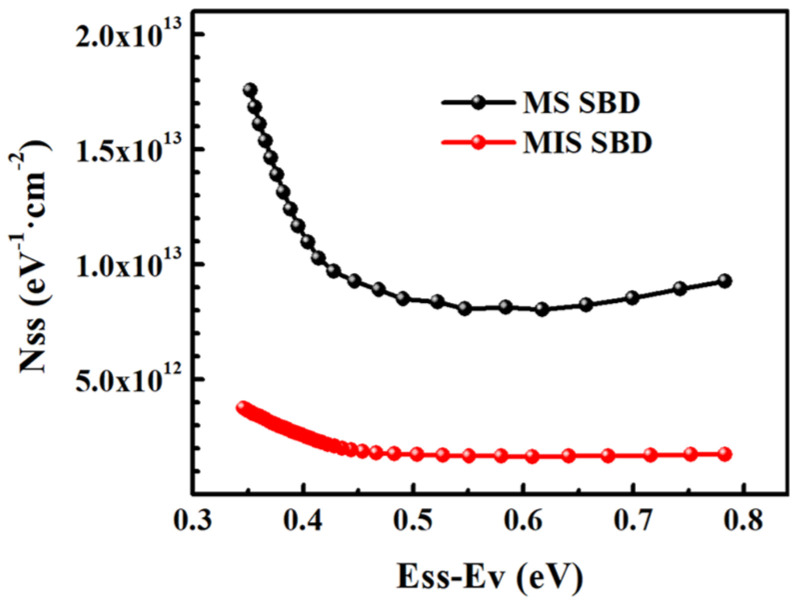
Energy distribution profiles of the *N*_SS_ of diamond MS SBDs without Al_2_O_3_ and MIS SBDs with Al_2_O_3_ determined from the I–V characteristics [125].

In 2021, Wang et al. introduced a 2 nm thick Al_2_O_3_ interfacial layer in the Zr/p-type diamond Schottky contact by the atomic layer deposition (ALD) technique. The inserted Al_2_O_3_ layer effectively passivated defects on the O-diamond surface, especially dangling bonds and oxygen vacancies, significantly reducing interface trap density [121,122,123]. For both SBDs with and without Al_2_O_3_ interlayers, as shown in Figure 8, *N*_SS_ values increase exponentially from midgap toward the top of the valence band. It is noticed that the *N*_SS_ values of MIS SBDs are significantly lower than those of MS SBDs, meaning that the number of dangling bonds decreases at the diamond surface after inserting the Al_2_O_3_ interlayer. With the incorporation of the Al_2_O_3_ layer, the Schottky barrier height increased from 1.356 eV to 1.694 eV, while the reverse leakage current was greatly reduced and the breakdown voltage improved from 60 V to 82 V. These enhancements were attributed to the interface passivation effect of Al_2_O_3_, which mitigates trap-assisted tunneling [124]. However, the insertion of the Al_2_O_3_ layer also resulted in a substantial increase in the series resistances, rising from 2.42 mΩ·cm^2^ to 15.14 mΩ·cm^2^. The larger resistance of the latter is caused by the resistive Al_2_O_3_ layer, even if it is quite transparent in carrier transport. Nevertheless, the interface quality and breakdown performance are improved, accompanied by an increase in series resistance to some extent.

Although the Al_2_O_3_ interlayer showed significant advantages in interface passivation, its moderate dielectric constant still limits its shielding capability under high electric field conditions, restricting further modulation of the interface band structure and SBH. In 2022, Zhang et al. [126] found that the introduction of an SnO_2_ insert layer also reduced *N*_SS_ effectively, while offering stronger field modulation due to its higher dielectric constant. This further increased the Schottky barrier height (from 1.45 eV to 1.84 eV), and the interface state density decreased by two orders of magnitude compared to the SBD without the SnO_2_ insert layer. Additionally, the device exhibited a higher breakdown voltage, increasing from 102 V to 123 V, demonstrating the effectiveness of the SnO_2_ insert layer in interface passivation. However, similar to Al_2_O_3_, the insertion of the SnO_2_ interlayer also leads to an increase in series resistance, rising from 0.67 mΩ·cm^2^ to 1.17 mΩ.·Consequently, in the forward I–V characteristics, the SBD with SnO_2_ exhibits a gradual deviation from linearity at higher bias, reflecting the impact of increased series resistance.

HfO_2_, a material with a high dielectric constant (*k* ≈ 25), would provide a stronger electric field shielding effect than Al_2_O_3_ and SnO_2_, effectively reducing the interface electric field [127]. In 2023, Han et al. [128] found that the introduction of the HfO_2_ insert layer effectively reduced the interface states, and the stability of the Schottky contact was optimized through its stronger electric field shielding effect. This interlayer further modulates SBH, significantly improving the rectifying performance and voltage with standing capability of the device. Specifically, after the HfO_2_ insert layer, the device’s barrier height increased from 0.75 eV to 0.99 eV, and *N*_SS_ decreased by nearly four times. It can be inferred that the interface quality is improved by decreasing the inhomogeneity of the O-diamond surface due to the well-passivated O-diamond surface [129,130]. Moreover, the MIS SBDs exhibited better rectifying characteristics, with the HfO_2_ insert layer significantly reducing reverse leakage current (from 10^−7^ A to 10^−9^ A), while the forward current and turn-on voltage showed slight decreases. Therefore, the HfO_2_ insert layer effectively suppressed the leakage current, demonstrating the dual role of the insert layer in optimizing the interface and modulating the barrier.

A comparative study of Al_2_O_3_, SnO_2_, and HfO_2_ insert layers reveals that these high dielectric constant materials play different roles in diamond SBDs. While Al_2_O_3_ and SnO_2_ primarily enhance the electrical properties of the device through interface passivation, HfO_2_ is able to provide a stronger electric field shielding effect compared to Al_2_O_3_ and SnO_2_ due to its high dielectric constant. While the incorporation of high-k interlayers effectively reduces *N*_SS_ and enhances breakdown voltage, this approach inevitably introduces a fundamental performance trade-off. These interlayers typically result in increased series resistance. Therefore, it is significant to carefully balance three competing factors, including *N*_SS_, breakdown voltage, and series resistance [131,132].

### 4.2. Barrier Modulation by Low-Work-Function Interlayers

Although interface passivation layers (such as Al_2_O_3_, SnO_2_, and HfO_2_) have greatly enhanced the performance of diamond SBDs and reduced *N*_SS_, further optimization of SBH remains a critical challenge. To this end, increasing attention has been directed toward employing low-work-function materials as interfacial interlayers.

LaB_6_, a novel low work function material (approximately 2.7 eV), has been introduced in the field of thermionic electron source [133,134,135]. In 2021, Shao et al. [136] first applied LaB_6_ as an interface insert layer in the Zr/p-type diamond Schottky contact structure. Low-work-function material, LaB_6_, acts as an electron donor [137], such that the electrons can flow from the LaB_6_ to the p-diamond and compensate the holes near the diamond surface, which is beneficial for formation of high quality Schottky contact. Compared to traditional metal/diamond structures without an interlayer, the SBH at the metal-diamond interface increased significantly from about 1.33 eV to 1.53 eV after introducing a 10 nm thick LaB_6_. The rectification ratio improved to 10^10^, and the reverse saturation current density decreased to 10^−19^ A cm^−2^ range. The breakdown voltage was significantly increased to 96 V. These improvements can be attributed to LaB_6_’s low work function, which modulates the SBH by altering the energy band alignment at the metal/diamond interface.

**Figure 9:**
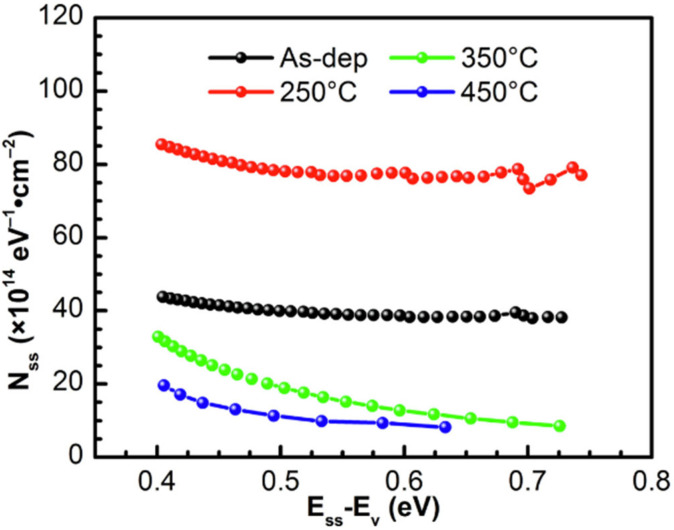
*N*_SS_ as a function of *E*_SS_ − *E*_V_ for as-deposited and RTA-treated diamond SBD with LaB_6_ [138].

Despite the improvement of the device performance by introducing a LaB_6_ insert layer, the ideality factor *n* remained as high as 1.85, indicating the presence of high interface state density. To address this issue, in 2023, Shao et al. [138] systematically studied the impact of rapid thermal annealing (RTA) on the interface quality of Zr/LaB_6_/p-type diamond SBDs under various annealing temperatures of 250 °C, 350 °C, and 450 °C, respectively. Figure 9 illustrates the *N*_SS_ as a function of energy (*E*_SS_ − *E*_V_) for samples subjected to different annealing treatments. As shown in the figure, the interface state density decreases significantly with increasing annealing temperature, especially at 350 °C. The study revealed that oxygen atoms migrated to the Zr/LaB_6_/diamond interface under the 250 °C annealing treatment, forming an inhomogeneous local oxide structure [139]. This results in an increase in the interface state density, which causes the increase in *n* and the slight reduction in Schottky barrier height. When the annealing temperature increased to 350 °C, the interface was improved, and the interface oxides were gradually transformed into a uniform and dense La-O, B-O, and ZrO_x_ composite interfacial layer. The 350 °C-annealed samples show a lower *n*, with the value of 1.78. The forward conduction characteristics of the device are improved. The interface resistance is significantly reduced (the series resistance is reduced to 0.79 mΩ·cm^2^), and the rectification ratio increased to 1.7 × 10^10^. The breakdown voltage remained at a high level of around 86 V, and the overall performance was excellent. However, when the annealing temperature was further increased to 450 °C, the LaB_6_ in the interfacial layer partially loses oxygen and decomposes, damaging the interfacial composite structure. This leads to the reactivation of interface states, reduction of the interfacial barrier, a sharp increase in the leakage current, and obvious degradation of the device performance.

In 2023, Zhu et al. [140] deposited 15 nm CeB_6_, another low work function material, on a p-type O-diamond SBD. The device structure was Zr/CeB_6_/diamond. Compared to the control sample of Zr/diamond without the insert layer, the SBH increased from 1.65 eV to 1.92 eV, the *n* decreased from 2.82 to 2.24, and the reverse breakdown voltage increased from −86 V to −110.5 V after the CeB_6_ insertion. Under the applied voltage equals ±10 V, the rectification ratio improved from 1.626 × 10^6^ to 1.503 × 10^7^, with a significant suppression of reverse leakage current. The optimization of the interfacial potential was further demonstrated by C–V measurements, where the width of the depletion layer was increased from 206.7 nm to 227.6 nm with a corresponding increase in the carrier concentration, verifying the optimization of the interfacial potential.

### 4.3. Terminal Electric Field Control Interlayers

**Figure 10:**
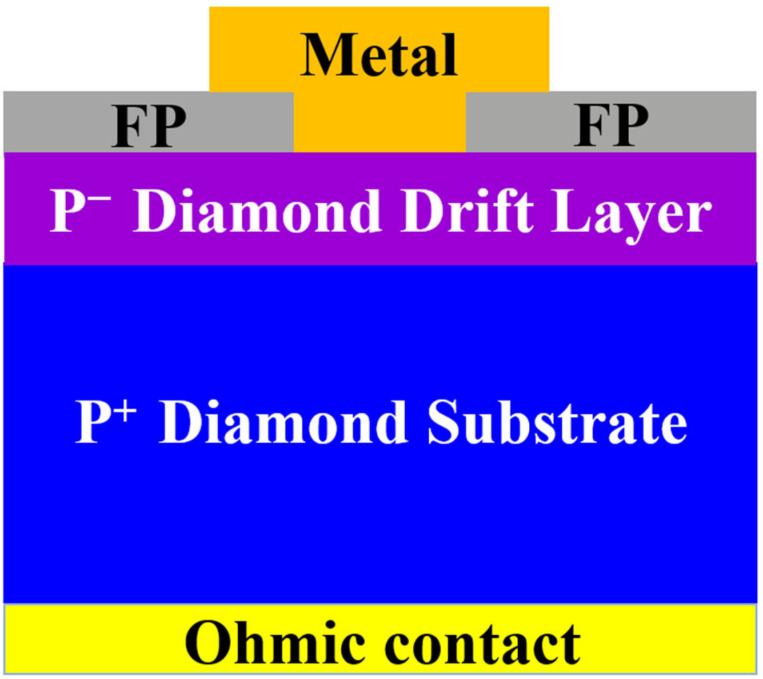
Schematic cross-sectional structure of the vertical p-diamond SBDs with field plate.

Although the interface insert layer technique has made significant progress in reducing *N*_SS_, optimizing SBH, and improving carrier injection efficiency, diamond SBDs still face the serious issue of concentrated terminal electric fields when high voltages are applied. This phenomenon is particularly pronounced at the edges of metal electrodes, where sharp electric field peaks can form, leading to premature electric field breakdown and causing the actual breakdown voltage to be much lower than the theoretical breakdown field strength of the material [141]. To further improve the breakdown voltage of diamond SBDs, researchers have introduced the field plate (FP) structure as a terminal engineering technique, as shown in Figure 10 [142,143]. A potential buffer zone is created at the electrode edge by extending the metal electrode edge and covering the electrode with a dielectric layer, which redistributes the edge electric field, shifts the breakdown location, and delays the breakdown behavior. This significantly improves the device’s breakdown voltage [144].

**Figure 11:**
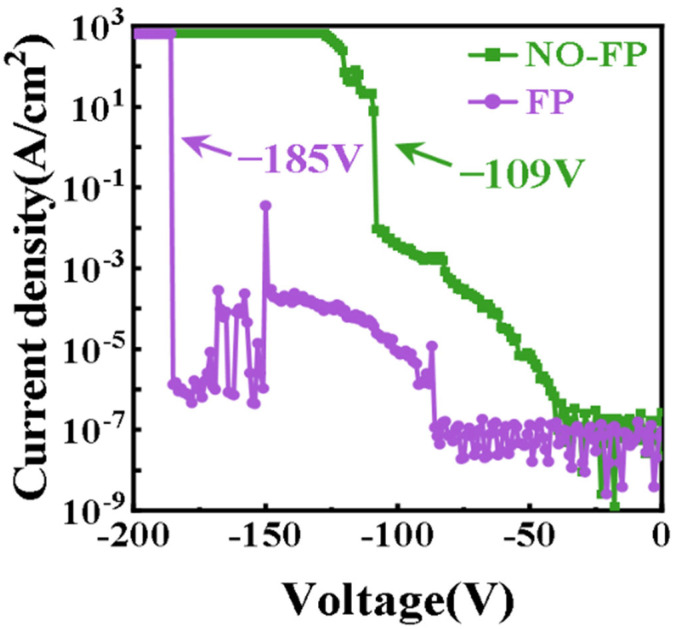
Reverse leakage current comparison of diamond SBDs with and without SnO_2_ FP structure [140].

In 2019, Zhao et al. [145] introduced a 500 nm thick SiN_X_ as the field plate in the Zr/Ni/Au diamond Schottky structure. The results showed that the reverse leakage current of the device decreased from 6.3 A/cm^2^ to 1.8 × 10^−6^ A/cm^2^, and the breakdown voltage was increased from 122 V to 133 V, while the edge electric field was improved from 3.2 MV/cm to 3.5 MV/cm. This indicates that the SiN_X_ field plate structure effectively smooths the abrupt potential gradient at the electrode edge, thereby mitigating electric field crowding and enhancing the overall field uniformity near the Schottky junction [146,147]. Thereafter, in 2022, Li et al. [148] used Al_2_O_3_ as the dielectric material for the field plate, which was subject to a thermal annealing treatment at 400 °C. The breakdown voltages of the diamond SBDs without and with annealing increased from 162 V to 386 V. The reverse leakage current of the latter was significantly reduced, and the forward voltage drop reduced from −2.3 V to −1.65 V. This reveals that the field plate of Al_2_O_3_ has significantly improved thermal stability and electric field buffering for diamond SBDs [149]. In the same year, Zhang et al. [140] proposed a novel FP structure using SnO_2_ as the dielectric material. This structure not only extends the electrode to modulate the edge electric field, but also takes advantage of the high dielectric constant of SnO_2_. As shown in Figure 11, the device with SnO_2_ FP exhibited a great improvement in breakdown voltage, increasing from 109 V (without FP) to 185 V (with FP), corresponding to a substantial suppression of reverse leakage current across the voltage range. The approach demonstrates the feasibility of incorporating high-k materials into FP structures for diamond SBDs.

In addition to traditional dielectric layer construction methods, Zhao et al. [150] also used C_4_F_8_ plasma treatment to form fluorination-terminated (FT) structure on the diamond surface, replacing C–O bonds with C–F bonds. Notably, this strategy did not rely on the extension of the electrode or additional dielectric layers deposition. Instead, it passively modulated the local electric field by the interlayer. As a result, the breakdown voltage increased from 97 V to 117 V, with the breakdown field reaching 3.3 MV/cm. The reverse leakage current significantly decreased, demonstrating the potential of surface termination modifications in field plate design [151].

The field plate structure, as a key technique for enhancing terminal breakdown capability in diamond SBDs, effectively addresses the common issue of edge electric field accumulation in traditional MS and MIS structures through dielectric coverage and edge potential modulation. Unlike interfacial insert layer structures, which focus on contact interface regulation, the field plate structure primarily affects the electric field in the terminal region of the device. Despite the demonstrated improvements in breakdown voltage through field plate engineering, it is worth noting that the experimentally achieved breakdown fields in diamond SBDs remain far below the theoretical value of ~10 MV/cm. This discrepancy arises from multiple factors. This discrepancy can be attributed to the presence of defects, which cause local electric field enhancement and induce premature breakdown [27]. In addition, the inhomogeneity of the Schottky barrier at the O-diamond interface results in localized regions with reduced barrier height. These low-barrier patches act as preferential conduction paths for reverse leakage current even under relatively low reverse bias, leading to early current rise and deviation from the ideal uniform-barrier model [91].

As shown in Table 1, although the introduction of FP structures effectively increases the breakdown voltage of diamond SBDs, other electrical parameters, such as the SBH and *n,* remain relatively unchanged or become worse. It suggests that while edge electric field suppression is beneficial for high-voltage operation, the FP structure does not directly affect the quality of the metal–semiconductor interface [152]. Therefore, further optimization of both the interfacial engineering and terminal field modulation is required to achieve a balanced improvement in all device parameters. Future optimization of the field plate structure can further integrate new material systems, such as two-dimensional materials, and leverage TCAD simulations for the quantitative design of terminal field distribution, supporting the stable operation of diamond power devices under extreme conditions.

In summary, the introduction of functional interlayers in diamond SBDs serves as an effective strategy to overcome the limitations posed by interface defects, Fermi level pinning, and poor carrier transport. Passivation layers with high-k dielectric layers (such as Al_2_O_3_, HfO_2_, SnO_2_) primarily function as interface passivation layers, effectively suppressing interface trap states, relieving electric field crowding through dielectric shielding, and enhancing both the SBH and breakdown voltage. In contrast, low work function materials such as LaB_6_ and CeB_6_ are utilized for barrier modulation. In addition, the field plate structure, as a means of controlling terminal electric fields, effectively suppresses edge electric field spikes through potential redistribution, enabling the device to operate stably in high-voltage environments. Collectively, these interlayers’ combined effects provide critical guidance for the future development of high-performance diamond SBDs.

## 5. Conclusions

Through the systematic review of the metal contact structures and interface engineering in diamond SBDs, it can be seen that the selection of single metals, the construction of multilayer and Interlayers show the decisive role of interface regulation on the enhancement of device performance. Due to their high work function and good stability, noble metals enable the formation of higher SBH and excellent thermal stability. Transition metals, with their moderate work function matching and processing compatibility with diamond interfaces, exhibit strong engineering practicality. Other metals, such as Zr, Al, and metal carbides (e.g., WC), offer extended solutions through their unique functionality and interface reactivity. On the other hand, multilayer-metal structures, through functional synergy between layers, effectively address various issues such as interface passivation, current uniformity, and diffusion stability, showing significant advantages under high-temperature and high-pressure conditions. Most significantly, MIS architectures represent a transformative approach in diamond SBDs’ development. By incorporating functional interlayers such as high-k dielectrics (Al_2_O_3_, HfO_2_), low-work-function materials (LaB_6_, CeB_6_), and field plate designs, MIS structures enable control of interface properties directly. These interlayers not only optimize interface states but also precisely modulate electronic band structures, leading to substantial improvements in SBH uniformity, leakage current suppression, and breakdown voltage enhancement.

Despite these advances, performance metrics associated with various metal and interface engineering methods suggest that SBH remains a key parameter for assessing the quality of metal/diamond contacts. However, it is important to recognize that the SBH values commonly reported in the existing literature are primarily derived from I–V measurements. In real devices, the main current paths in the device tend to be concentrated in the regions with low localized potential barriers rather than the average potential barrier at the interface. As a result, the I–V approach underestimates the true SBH. Thereby, it is significant to establish a unified extraction framework that combines I–V and C–V analysis with an appropriate model for accurately assessing interface properties. Such standardized evaluation protocols will enable comprehensive performance and accelerate the practical implementation of diamond SBDs in next-generation power electronics.

Moreover, it should be emphasized that the device performance is not solely determined by the metal and interlayer design. The crystalline quality of the epitaxial diamond layer, the surface condition of the substrate, and the deposition technique used for interlayers all play essential roles in SBDs. A holistic optimization of all these factors is necessary to fully realize the potential of diamond-based SBDs.

Although diamond devices are still in the research phase, the material’s superior properties, such as an ultra-wide bandgap, extremely high thermal conductivity, and bulk carrier mobility, make it very promising among WBG semiconductors. Thus, it is of great value to continue exploring diamond-based technologies, while drawing lessons from the successful development paths of SiC and GaN devices.

## Figures and Tables

**Table 1:** Summary of the parameters of the fabricated diamond SBDs and reported parameters.

Material	FP	SBH (eV)	*n*	BV (V)	Ref
HfO_2_	w/	1.55	1.48	280	[153]
	w/o	1.57	1.46	183	
Al_2_O_3_	w/	\	\	1800	[154]
	w/o	\	\	900	
SiN_x_	w/	1.6	2	133	[145]
	w/o	1.8	1.6	122	
SnO_2_	w/	1.65	1.31	185	[140]
	w/o	1.66	1.30	109	
SiO_2_	w/	0.47–1.44	1.02–1.52	407	[152]
FT−Diamond	w/	0.82–1.44	2.12–2.66	97–117	[150]
	w/o	0.69–1.83	1.55–3.55	73.5–85	
